# COVID-19-related social isolation and symptoms of depression and anxiety in young men in Poland: Does insomnia mediate the relationship?

**DOI:** 10.1371/journal.pone.0285797

**Published:** 2023-05-18

**Authors:** Justyna Mojsa-Kaja, Klaudia Szklarczyk-Smolana, Ewa Niedzielska-Andres, Anna Kurpińska, Joanna Suraj-Prażmowska, Maria Walczak

**Affiliations:** 1 Department of Neurobiology and Neuropsychology, Institute of Applied Psychology, Jagiellonian University, Krakow, Poland; 2 Chair and Department of Toxicology, Jagiellonian University Medical College, Krakow, Poland; 3 Jagiellonian Centre for Experimental Therapeutics, Jagiellonian University, Krakow, Poland; Sapienza University of Rome, ITALY

## Abstract

The need for physical distancing due to COVID-19 mitigation efforts forced prolonged social isolation, which may affect sleep and lead to mental health problems. Previous research has shown that young adults are particularly vulnerable to psychological stress caused by social isolation, the negative psychological impact of the pandemic, and greater frequency and severity of sleep problems. Therefore, the main goal of the present study was to examine whether insomnia could constitute a mediation mechanism that explains the relationship between social isolation experienced during the COVID-19 pandemic and mental health outcomes (depression and anxiety) reported up to 1.5 years later. The study was conducted among young (M±SD; 24.08±3.75) men (N = 1025) in Poland. Data were collected by means of self-report questionnaires, including *The Social Isolation Index*, *The Athens Insomnia Scale*, *The State-Trait Anxiety Inventory (STAI-S)* and *Beck’s Depression Inventory (BDI-II)*. The results show that insomnia mediates the relationships between social isolation and both anxiety and depression. The current findings emphasize the role of insomnia in the relationships between social isolation experienced during COVID-19 and negative emotional states. From a clinical perspective, the results suggest that implementing therapeutic components that address social isolation in insomnia treatment programs may prevent the development of depression and anxiety symptoms among young men.

## Introduction

Next to climate change, depletion of natural resources, and wars, the COVID-19 pandemic negatively affects our mental health and is one of the greatest challenges of our time [1]. Daily worries related to these 21st-century issues are concerning in terms of the psychological maladjustment and impaired well-being of those who experience individual distress.

In the wake of the declaration of the COVID-19 pandemic, social isolation and lockdown [2] were some of the main concerns due to their potentially dangerous consequences, such as the likelihood of developing symptoms of depression and/or anxiety [3, 4]. To prevent transmission of the virus and limit the outbreak, governments and public health systems adopted extraordinary measures, which changed the daily routines and social relationships of millions of people. In March 2020, the Polish government declared a state of epidemic threat throughout the country [5]. This was followed by a series of regulations and restrictions, including the closure of schools, universities, cultural institutions, restaurants, and non-essential shops. The lockdown regulations also included a ban on gatherings of more than two people, a requirement for people to cover their mouths and noses in public places, and a limit on the number of people allowed in shops and on public transport. There were also restrictions on international travel, with borders closed to non-essential travel and mandatory quarantine for those arriving from outside the country.

The restraint strategies in response to the global COVID-19 pandemic have impacted various lifestyle habits, such as social relationships, mobility, and sleep, with implications for mental health [6–10]. The need for physical distancing due to these coronavirus mitigation efforts forced the prolonged social isolation and loneliness of both young and old adults [11]. Social isolation is an objective state marked by little or infrequent social contact, while loneliness is the subjective feeling of social isolation, often defined as the discrepancy between the actual and desired level of social connection [12]. Both, social isolation and loneliness are related constructs that denote a degree of social disconnection.

Social isolation, as a source of stress, can affect sleep [13]. Stressors play a crucial role in the pathogenesis of sleep disturbances [14]. During times of stress, existing sleep problems may increase and new ones may emerge [15]. The COVID-19 pandemic became a major stressor for millions of people, with some researchers suggesting that it could even be a source of traumatic stress [16]. Thus, the escalation of sleep problems such as insomnia is considered a common negative consequence of the stress evoked by the pandemic [17]. Given the high rates of acute insomnia associated with the COVID-19 pandemic, which has proved to be a precursor of psychiatric disorders [18], individuals who develop sleep disturbances during COVID-19 may be at greater risk of long-term detrimental consequences. Recently, Morin et al. [19], documented the prevalence of clinical cases of insomnia (36.7%), anxiety (25.6%), and depression (23.1%) during the COVID-19 pandemic in 13 countries throughout the world. The study showed that the risk of insomnia was higher among younger age groups and among participants who were living alone and were thus socially isolated. These data suggest that stressful life events (such as social isolation) may play–particularly in a group of young adults–a crucial role in the pathogenesis of insomnia, which leads to anxiety and depression. Thus, describing the mechanisms of the relationships between psychological distress caused by social isolation and insomnia and mental health problems may be beneficial for both scientific purposes as well as educational and therapeutic interventions.

Understanding these associations in young adults is uniquely important. Research conducted during the COVID-19 pandemic showed that nearly half of people between the ages of 19 and 29 years have reported feeling symptoms of anxiety or depression at a significantly higher rate compared to other age groups [20]. Younger age predicted not only a negative psychological impact of the COVID-19 pandemic but also greater frequency and severity of sleep problems [17]. Additionally, according to Beam and Kim [21], young adults are particularly vulnerable to psychological distress caused by social isolation; in comparison to other populations, they are far more likely to seek intervention due to the psychological effects of self-isolation and loneliness. Finally, compared to women, young men are especially prone to experiencing a feeling of solitariness and higher levels of emotional distancing [22]. Young men reported higher levels of loneliness during the COVID-19 pandemic than young women, and this was associated with worse psychological and physiological outcomes [23]. Although women are more concerned about the negative health consequences of COVID-19, young men are more likely to experience such consequences [24].

Overall, while there is not a large body of research on this specific topic, the available evidence suggests that young men may have had a harder time coping with COVID-19-related isolation than women. The aim of the study was to examine the relationship between lockdown-related social isolation, insomnia, and mental health problems in the young male population in Poland.

It should be underlined that insomnia is both a common symptom of and risk factor for a range of psychiatric disorders, including anxiety and mood disorders. Thus, although the relationship between sleep problems and mood disorders is complex and likely bi-directional, sleep problems precede depression to some extent [25]. Thus, we hypothesize that insomnia mediates the relationship between the stress of experienced social isolation during the COVID-19 pandemic and negative outcomes such as depression and anxiety among young male adults.

## Participants

The data were gathered in January 2022 in Poland. The research was conducted with the use of a professional research panel that has access to a total sample of 280 000 respondents, including 32 000 young male respondents (18–30 years old), from which the study sample was randomly selected. The calculation of the sample size was based on the following assumptions: a 95% (0.95) confidence level and a fraction size of 0.5 with a maximum estimation error of 4%. The reference population was 2 691 189. One thousand and twenty five male respondents (age: M = 24.08; SD = 3.75) participated in a voluntary web-based study. Table 1 contains the sociodemographic characteristics of the study participants.

**Table 1:** Sociodemographic characteristics of the male study participants (N = 1025).

Variable	N (%) of participants
**Age**	
18–23 years	461 (44.97)
24–30 years	564 (55.02)
**Education**	
Low level	168 (16.39)
Medium level	560 (54.63)
High Level	297 (28.97)
**Marital status**	
Single	570 (55.61)
Informal relationships	300 (29.27)
Married	147 (14.34)
Widow	2 (0.20)
Divorced	6 (0.59)
**Locality**	
Urban	773 (75.41)
Rural	252 (24.59)

## Methods

The current study focused on the construct of social isolation. A social isolation index was constructed from three questions; this index is the same as the social isolation index used by UK Biobank [26]. The questions were translated into Polish with the use of a back-translation procedure. The participants were asked to answer questions related to social isolation in the March–May 2020 period.

Question 1: “Including yourself, how many people were living together in your household during the LOCKDOWN due to the COVID-19 pandemic (March–May 2020)?” (1 point was given for living alone).

Question 2: “How often did you visit friends or family or had them visit you during the LOCKDOWN due to the COVID-19 pandemic (March–May 2020)?” (1 point was given for answering about once a month, once every few months, never or almost never, or no friends or family outside household);

Question 3: “Which of the following (sports club or gym, pub or social club, religious group, adult education class, other group activity) did you engage in once a week or more during the LOCKDOWN due to the COVID-19 pandemic (March–May 2020)?” (1 point was given for answering none of the above).

Individual scores were summed to calculate an overall score ranging from 0 to 3.

*Insomnia* was measured by the Polish version [27] of the 8-item Athens Insomnia Scale [28], which measures the severity of insomnia based on the ICD-10 criteria (e.g., "Awakenings during the night"; α = .82). Items are rated on a 4-point scale (from "no problem at all’’ to "very serious problem").

### Depression

As is consistent with the original version of BDI-II [29], the Polish version [30] contains 21 items on a 4-point scale from 0 (symptom absent) to 3 (severe symptoms) and is a 21-item self-report inventory for evaluating the severity of depression in normal (α = .91) and clinical populations (α = .93). It assesses depressive symptoms within the preceding week, with high scores reflecting greater severity of depressed mood. Total scores range from 0 to 63.

### Anxiety

The Polish version [31] of the State-Trait Anxiety Inventory [32], was used to determine state of anxiety. STAI-S is a 20-item questionnaire, most frequently used for measuring the state of anxiety in psychological research studies due to its psychometric properties (α = 0.94).

All participants were treated in accordance with the ethical guidelines of the Helsinki Declaration. The participants were provided with information about the aim of the study; they provided their written informed consent to participate and were informed about the possibility of withdrawing from the study at any stage. The study was accepted by the Research Ethics Committee of the Institute of Applied Psychology, Jagiellonian University. All data were anonymized and are used in aggregate; no personal identifiers are reported.

## Results

Data were analyzed using SPSS v.26 and AMOS v.26.

Relevant tests were carried out to examine the probable existence of multicollinearity. The degree of multicollinearity of the study variables was found to be acceptable, with all tolerance values shown to be higher than 0.96, and the VIF values are lower than 1.01.

Table 2 shows descriptive statistics and intercorrelations among the study variables.

**Table 2:** Descriptive statistics of the variables and correlations between social isolation, insomnia, depression and anxiety (N = 1025).

Variable	M	SD	Correlation			
			1	2	3	4
1. social isolation	1.26	.82	–	.07*	.14**	.16**
2. insomnia	7.01	4.71		–	.65**	.60**
3. depression	13.14	11.47			–	.71**
4. anxiety	43.61	11.81				–

*p < 0.05

** p <0.01.

Social isolation was correlated with insomnia (r = .07, p < .05) and both depression (r = .14, p < .01) and anxiety (r = .16, p < .01). Additionally, insomnia correlated with depression (r = .65, p < .01) and anxiety (r = .60, p < .01).

Over 40% of participants reported above-normal depression symptoms; 49.36% reported above-normal anxiety symptoms; and 55.9% reported above-normal insomnia. Moreover, 42.63% of participants reported severe isolation, and some had experienced severe levels of depression (11.7%), anxiety (49.36%) and insomnia (4.39%) during the COVID-19 pandemic (Table 3).

**Table 3:** Levels of social isolation, insomnia, depression and anxiety symptoms (N = 1025).

Social isolation	Participants (%)	Insomnia	Participants (%)	Depression	Participants (%)	Anxiety	Participants (%)
0–least	20.48	0–5 normal	44.09	0–13 minimal	59.14	20–30 low	15.41
1–moderately	36.87	6–9 mild	27.41	14–19 mild	14.43	31–43 average	35.22
2–3 most	42.63	10–15 moderate	24.09	20–28 moderate	14.73	44–80 high	49.36
		16–24 severe	4.39	29–63 severe	11.70		

The significant correlations between isolation, insomnia, depression and anxiety (Table 2) enabled the following mediation models to be tested:

1. the mediating role of insomnia on associations between isolation and depression;
2. the mediating role of insomnia on associations between isolation and anxiety.

The direct and indirect (via the mediator) effects of isolation on depression and anxiety were calculated using a bootstrap estimation technique with 1,000 samples. If the confidence intervals did not include zero for an indirect effect, results were considered significant.

All analyzed indirect effects were significant (Table 4). Insomnia mediated the effect of isolation on depression and anxiety. The indirect-to-total effect ratios show that insomnia explained (1) 32% of the relationship between isolation and depression, and (2) 41% of the relationship between isolation and anxiety.

**Table 4:** Effects of isolation on depression and anxiety mediated by insomnia.

Dependent variable	Effect					95% CI	indirect/total ratio
	IV on M	M on DV	direct: IV on DV	total: IV on DV	indirect: IV on DV via M		
Depression	.07*	.65*	.10**	.14**	.045*	.005–.089	.32
Anxiety	.07*	.60**	.12**	.10**	.041*	.006–.081	.41

IV–independent variable; DV–dependent variable; M–mediator; CI–confidence intervals; indirect/total ratio–ratio of the indirect effect to total effect (c′) indicating a magnitude of mediated effect.

*p < 0.05

** p <0.01.

## Discussion

Although COVID-19-related restrictions are legitimate under a time of global crisis, they can lead to social isolation, which in turn has a large negative effect on the mental health of older adults [33], and it has an even larger effect on adolescent and young adult populations [21]. The strongest association between social isolation and mental illness was between depression and anxiety, a finding that was consistent across studies of adolescents and young adults [34]. Moreover, sleep problems were associated with anxiety and depression among individuals who self-isolated during the pandemic [35, 36]. Thus, in line with previous research conducted during the COVID-19 pandemic, we focused on analyzing the relationship between social isolation experienced in times of pandemic, insomnia, anxiety, and depression among young men.

As reported by respondents during the COVID-19 pandemic, we found that 20.48% experienced mild social isolation, 36.87% experienced moderate isolation, and 42.63% experienced severe isolation. In pre-pandemic research among men, Smith et al. [26] reported that 44% had experienced mild social isolation, 40.1% had experienced moderate isolation, and 15.9% had experienced severe social isolation, but severe isolation during the COVID-19 pandemic reached even higher levels than this.

We found a tendency of increased insomnia, depression and anxiety levels, which is congruent with a recent pandemic study by Morin et al. [19]. We analyzed the interrelationships between the described constructs; as a result, we connected four variables: social isolation predicted insomnia, which in turn predicted higher depression and anxiety. However, it should be emphasized that the correlation between retrospectively measured social isolation and currently reported insomnia is relatively low (0.07) but still significant and congruent with previous findings, thus showing that people who experience social isolation develop insomnia, which, in turn, is an important predictor of mental disorders, including anxiety disorders and depression [37]. Moreover, as was shown in previous research [38], even periods of isolation that last less than 10 days can have long-term effects, and psychiatric symptoms can persist for up to 3 years. Thus, we have shown that the social isolation experienced during the COVID-19 lockdown might have consequences 1.5 years later in the form of insomnia and further disorders, such as depression and anxiety. To the best of our knowledge, this is the first investigation to present a meditation mechanism of insomnia that explains the relationship between the stress of experienced social isolation during the COVID-19 pandemic and both depression and anxiety among young male adults. These data suggest that stressful conditions related to social isolation as a result of the pandemic may induce mental health disturbances through insomnia.

One possible interpretation for the link between social isolation, insomnia, depression, and anxiety may be the shared biological mechanism of activation of the hypothalamic-pituitary-adrenal (HPA) axis. Human and animal investigations of neuroendocrine stress mechanisms suggest that social isolation increases activation of the HPA axis [39]. Similarly, dysfunction of the HPA axis is implicated in the pathogenesis of insomnia [40], which has been considered a physiological “hyperarousal” disorder, reflected by HPA axis activation [41]. Moreover, research findings point to the possibility of HPA axis overactivity in insomnia and both depression [42] and anxiety [43]. Thus, stressful life events such as social isolation make individuals more vulnerable to insomnia [44] via activation of the HPA axis, which increases the likelihood of anxiety and depression [45].

The role of insomnia in the relationship between social isolation and a higher risk of depression and anxiety has important clinical implications. It has previously been suggested that treating insomnia might alleviate many mental health problems; furthermore, addressing sleep disturbances at their earliest stages might in fact prevent the onset of some clinical mental health disorders [46].

Thus, from a clinical perspective, the present findings suggest that addressing social connectedness could contribute to reducing stress [47] and preventing the development of depression and anxiety [48]. Implementing therapeutic components in insomnia treatment programs, such as social skills training, would be useful to enhance social connectedness. Additionally, emphasizing treatment modalities such as enhancing social support, increasing opportunities for social contact, and addressing maladaptive social cognition [49] could help reduce social isolation and diminish the prevalence of insomnia, anxiety and depression among young men.

The impact of COVID-19-related isolation on mental health is complex and multifactorial, and it is important to consider potential co-existing factors that may affect it. Other 21^st^-century challenges, like climate change-related disasters, economic downturns, and political instability may all influence the relationship between isolation and mental health [1]. However, it is also possible that COVID-19 isolation due to lockdown may have a unique mental health impact that requires targeted treatment [50, 51]. Therefore, in order to provide effective support and resources to those in need, public health interventions aimed at mitigating the negative effects of isolation on mental health should take into account not only the specific cause of isolation but also the confounding factors.

The study’s findings suggest several future prospects for public health interventions aimed at mitigating the negative mental health effects of COVID-19-related isolation. Mental health screening protocols could be updated to include questions about sleep quality and insomnia as these factors may be important indicators of an individual’s wellbeing after periods of isolation. The study highlights the critical role that sleep plays in mental health. Public health campaigns and educational initiatives could be developed to increase awareness of the importance of sleep and the negative effects of poor sleep quality on mental health, especially among young men. The importance of social connections among young people must also be taken into account since young adults experienced more negative effects of isolation on mental health than older ones [52, 53]. Future studies and social policies should consider age differences in order to offer an approach that is tailored to the specific needs of each age group. By promoting mental health screening, targeting insomnia, and raising awareness of the importance of sleep, public health efforts could help prevent and treat depression and anxiety resulting from periods of isolation, especially among young men.

The present study has several limitations. First, the data is correlational in nature. Although we argue that social isolation (measured in a retrospective manner) precedes insomnia, depression and anxiety (measured 1.5 years after the analyzed period of lockdown), all variables were assessed at the same time and causal claims cannot be made without further longitudinal research. Thus, using a longitudinal design in future research may be adequate to assess the causal relationship between social isolation, insomnia, anxiety, and depression.

Secondly, the retrospective assessment of social isolation might have introduced some memory bias. It is well known that respondents tend to give less accurate answers when asked about the past than when asked about the present. Thus, being aware of the drawbacks and limitations of a retrospective survey related to the COVID-19 pandemic, we have tried to reduce the memory bias by following [54], who suggested minimizing the cognitive effort associated with retrospective questions by using short, understandable questions that require respondents to provide objective facts that prompt higher recall accuracy than subjective evaluations. Furthermore, using specific anchor points (such as a precisely determined period of time March–May 2020) could have helped respondents to recall objective facts so they would provide accurate answers about their social contact during COVID-19 lockdown.

Thirdly, as has previously been noted, respondents tend to report past experiences and feelings that are more consistent with their current situation [54], and depressed mood could influence the retrieval of information [55]. Thus, individuals’ levels of depression, as assessed in 2022, might have influenced their retrospective assessment of the social isolation experienced during the COVID-19 lockdown (March–May 2020).

Moreover, we have focused purely on objective social isolation, with no focus on the association between loneliness related to isolation. Further investigation should analyze the link between those two constructs and both insomnia and negative emotional states.

Additionally, the sample was composed of a homogenous group of young men, which makes comparisons impossible regarding gender or age differences in the analyzed relationships; thus, the result may not be generalizable to general populations. The lack of women in the study limits the applicability of the study’s conclusions. Women may be less or more likely to have mental health issues resulting from lockdown-related isolation, and women’s experience of mental health issues may be different from that of men. Therefore, it would be important to include women in further studies to ensure that the findings are representative of the entire population and are not biased towards men. Moreover, previous studies have indicated that Athens Insomnia Scale is useful in the assessment of the subjective symptoms of insomnia [56] during the COVID-19 pandemic; however, including objective measurements such as polysomnography and actigraphy in future studies may help to measure sleep deprivation more objectively.

Although the measure of social isolation used in the UK BioBank study has been widely used and cited, to the best of our knowledge it has not been previously psychometrically validated. Further research on transcultural validation and in-depth verification of its psychometric properties is required, and the lack of psychometric characteristics constitute a limitation of the current study.

Finally, there is a substantial body of pre-pandemic research analyzing the links between social isolation, insomnia and mental health problems [57, 58]. Thus, the examined associations between the examined constructs (social isolation, insomnia, depression, and anxiety) may be observed regardless of the COVID-19 pandemic. Although further research is needed to address the contextual role of the pandemic, we suggest social isolation during a pandemic is a crucial stressor that leads to insomnia and, in consequence, to depression and anxiety.
